# EMMPRIN deficiency alleviated metabolic-associated steatohepatitis progression via regulation of the UBA52–MCT1 axis

**DOI:** 10.3389/fphar.2026.1706859

**Published:** 2026-01-23

**Authors:** Linying Lai, Baoyuan Huang, Ziping Song, Xinyan Zhu, Wenzhuo Yang

**Affiliations:** 1 Department of Gastroenterology and Hepatology, Tongji Hospital, School of Medicine, Tongji University, Shanghai, China; 2 Department of Gastroenterology, Shanghai East Hospital, School of Medicine, Tongji University, Shanghai, China

**Keywords:** EMMPRIN, MASH, MCT1, UBA52, ubiquitination

## Abstract

**Background:**

Metabolic dysfunction-associated steatohepatitis (MASH) is characterized by a lipid overload-induced pathological cascade featuring hepatocyte injury, inflammation, and progressive fibrosis. This study aims to systematically investigate the role of EMMPRIN in MASH progression, and to elucidate its mechanisms in reprogramming the hepatic metabolic microenvironment.

**Methods:**

Murine models induced by methionine-choline -deficient diet, hepatocyte-specific EMMPRIN overexpression and knockout mice models were used to evaluate EMMPRIN’ roles in steatohepatitis. Parallel *in vitro* studies were conducted in corresponding cellular models. Proteomic sequencing, mass spectrometry, co-immunoprecipitation, Western blotting, quantitative PCR, and immunofluorescence were employed to identify downstream targets and characterize ubiquitination modifications.

**Results:**

EMMPRIN overexpression significantly exacerbated MASH phenotypes, including hepatic steatosis, inflammatory infiltration, and collagen deposition. Conversely, EMMPRIN knockout conferred substantial protection against these pathological changes both *in vivo* and *in vitro*. Mechanistically, EMMPRIN downregulated UBA52 expression, resulting in reduction in the free ubiquitin pool and subsequent decrease in K63-linked polyubiquitination of monocarboxylate transporter 1 (MCT1). This ubiquitination defect led to destabilization of MCT1 and was associated with a global increase in protein lactylation in EMMPRIN-deficient models. Furthermore, EMMPRIN suppression inhibited several signaling pathways critically involved in MASH pathogenesis, including PPAR signaling, Notch signaling, and TGF-β-mediated fibrotic response.

**Conclusion:**

Our findings demonstrate that EMMPRIN promotes MASH progression through the UBA52-MCT1 regulatory axis, which modulated ubiquitin-dependent protein stability and induced metabolic reprogramming, thereby driving lipid accumulation, inflammation, and fibrosis. These results position EMMPRIN as a promising therapeutic target for MASH intervention.

## Highlights

- EMMPRIN drove MASH progression by exacerbating steatosis, inflammation, and fibrosis *in vivo* and *in vitro*.
- EMMPRIN impaired K63-linked ubiquitination and stability of MCT1 via UBA52 suppression and triggered lactylation-mediated metabolic reprogramming.
- EMMPRIN knockout conferred hepatoprotective effects and suppressed pro-fibrotic and metabolic signaling pathways.
- Targeting the EMMPRIN-UBA52-MCT1-lactylation axis represented a novel therapeutic strategy for MASH.

## 1 Introduction

With the shifting global landscape of liver diseases, metabolic dysfunction-associated steatotic liver disease (MASLD) has emerged as a condition of epidemic proportions, exhibiting the most rapid increase in both incidence (38.2%) and mortality (17.05/1,000 person-years) among liver diseases (Marchesini et al., 2024; Younossi et al., 2023). Current epidemiological projections indicate that by 2040 (Le et al., 2022), its global prevalence is expected to exceed 55%, posing a substantial public health challenge worldwide. MASLD progresses along a well-defined clinical spectrum, advancing from simple steatosis to aggressive metabolic dysfunction-associated steatohepatitis (MASH), which currently affects approximately 5% of the general population globally and nearly 16% of individuals with pre-existing MASLD (Younossi et al., 2023). If left untreated, MASH can progress to hepatic fibrosis, liver failure, and even hepatocellular carcinoma. Beyond its hepatic manifestations, MASLD demonstrates strong associations with extrahepatic complications, including cardiovascular and cerebrovascular diseases, and has become a leading indication for liver transplantation in many regions (Huang et al., 2025). The therapeutic landscape for MASH is undergoing rapid transformation. The landmark approvals of resmetirom (Rezdiffra) and, more recently, semaglutide (Wegovy) have provided the first FDA-approved pharmacotherapies specifically indicated for this condition (Bansal et al., 2025; Lazarus et al., 2024; Noureddin et al., 2024). These breakthroughs represent significant milestones in the field, offering hope for effective disease modification. Despite these advances, therapeutic options within the clinical armamentarium remain limited and efficacy is often only partial, driving ongoing research into novel disease mechanisms and the development of more effective targeted therapies.

EMMPRIN (Extracellular Matrix Metalloproteinase Inducer), also known as Basigin, is a key transmembrane glycoprotein involved in a variety of physiological and pathological processes, including inflammation, wound healing, extracellular matrix remodeling and tumor progression (Asgari et al., 2023; de la Cruz Concepción et al., 2022; Guindolet and Gabison, 2020; Zhu et al., 2017). Accumulating evidence suggests that EMMPRIN plays a significant role in metabolic and inflammatory disorders. For instance, studies have shown that intracellular hyperglycemia may induce abnormal glycosylation of EMMPRIN, enhancing its MMP-inducing activity and contributing to the pathogenesis of diabetes (Mahmoud and Ali, 2021). Furthermore, downregulation of EMMPRIN has been identified as an important mechanism by which H_2_S stabilizes atherosclerotic plaques and suppresses inflammatory signaling (Munteanu et al., 2024). EMMPRIN produced by activated monocytes was also reported to be a novel inflammatory mediator and represented a potential therapeutic target in psoriasis (Amezcua-Guerra et al., 2020). Interestingly, EMMPRIN also exhibits anti-fibrotic properties in certain contexts, as it has been shown to inhibit keloid formation by suppressing Smad2 signaling and reducing fibronectin expression by suppressing the Smad2 signaling and reducing fibronectin expression (Diao et al., 2025). Conversely, in HBV-related liver fibrosis, its expression is directly upregulated by the TGF-β1/Smad4 pathway, thereby promoting fibrotic progression (Li et al., 2015). Given that metabolic dysregulation, chronic inflammation, and fibrosis represent the central pathological features of MASH, we hypothesize that EMMPRIN may play a critical role in the development and progression of this condition.

Therefore, to systematically investigate the functional role of EMMPRIN in MASH pathogenesis, we established hepatocyte-specific EMMPRIN overexpression and deletion mouse models, along with corresponding *in vitro* EMMPRIN knockdown cell lines. Using these systems, we performed comprehensive proteomic sequencing and mass spectrometry analysis to delineate the regulatory network governed by EMMPRIN in MASH. Our results demonstrated that EMMPRIN exacerbates MASH progression by coordinately modulating metabolic dysregulation, inflammatory activation, and fibrotic responses. Further mechanistic studies identified monocarboxylate transporter 1 (MCT1) as a key target through which EMMPRIN promotes MASH development. Given the established role of ubiquitination in regulating protein stability and signaling pathways (Zhang et al., 2025). We further elucidated that EMMPRIN regulated MCT1 function via a ubiquitin-dependent mechanism, uncovering a novel regulatory axis contributing to MASH progression.

## 2 Materials and methods

### 2.1 Cell culture and treatment

Under the condition of 5% CO2 and 37 °C, human THLE-2 cell purchased from Fuheng biology (Shanghai, China) was cultured according to the manufacturer’s instructions in Dulbecco’s Modified Eagle Medium supplemented with 10% fetal bovine serum (FBS) and 1% penicillin-streptomycin solution. Cells were treated with 1 mM long chain fatty acid (FFA) (Oleate: Palmitate at ration of 2:1, O1383, P0500, Sigma-Aldrich, St. Louis, MO, United States) in 1% bovine serum albumin (A8020, Solarbio, Beijing, China) for 48 h to establish an *in vitro* model of control and steatosis hepatocyte (Li et al., 2024; Park et al., 2020; Yan et al., 2022). The small interfering RNAs (siRNAs, RiboBio) and plasmids (Genechem Co., Ltd. and Miaoling Biotechnology Co., Ltd.) were transiently transfected by Lipofectamine 3000 (L3000015, Invitrogen, Carlsbad, CA, United States) according to the manufacturer’s instructions.

### 2.2 Cell viability assay

The impact of FFA loading on THLE-2 cells was evaluated by measuring cell viability with the CCK-8 assay. Briefly, 5,000 cells were seeded in 96 well plates and allowed to adhere overnight. After with/without FFA treatments for 0, 24, 48, 72 h, 10 μL of CCK8 solution was added in each well and incubated for 1 h at 37 °C, and absorbance at 450 nm was measured, adhering strictly to the protocol outlined by the kit’s manufacturer (C6005, NCM Biotech). The growth curves of each group of cells were plotted on GraphPad prism software based on the results of experiments.

### 2.3 Animals and treatment

All animal experiments were approved by the Animal Research Ethics Committee of Tongji Hospital (Shanghai, China). C57BL/6J mice (6–8 weeks old) were purchased from Huachuang Sino (Jangsu, China). Mice were housed under pathogen-free conditions on a 12 h light/12 h dark cycle at 22 °C. After a 2 week acclimatization period, C57BL/6J mice were randomly assigned two groups: AAV8-EMMPRIN^Ctrl^ and AAV8-EMMPRIN^OE^. For AAV8-mediated EMMPRIN overexpression (Cyagen Biosciences Inc. Suzhou), 200 μL of adeno-associated virus was administered via tail vein injection. The TBG promoter was used to drive hepatocyte-specific expression of the AAV8 vector. EMMPRIN^flox/flox^ (EMMPRIN^f/f^) mice on a C57BL/6J background were generated by Cyagen Biosciences (Suzhou, China) using the CRISPR/Cas9 system. Two sgRNAs (gRNA1# and gRNA2#) were designed to target intronic regions flanking exons 2–8 of EMMPRIN, enabling loxP site insertion via a homology-directed repair template. Founders carrying the floxed allele were identified by PCR screening. Hepatocyte-specific EMMPRIN knockout mice (here after referrd to as EMMPRIN^Cre^) were subsequently generated by crossing EMMPRIN^f/f^ mice with Alb-Cre mice (Jackson Laboratory, United States). Mice were randomly assigned to experimental groups during the study. Mice were fed a methionine/choline-deficient (MCD) diet (TP36226MCD/G, Trophic, China) for 12 weeks to induce fatty liver disease. After the feeding period, liver tissues were then collected for protein extraction, sectioning, and histological staining following humane euthanasia under anesthesia for subsequent analysis. The mice were anesthetized with isoflurane of 2% inhalation before sacrifice, and euthanasia was subsequently performed by cervical dislocation under deep anesthesia.

### 2.4 Histological analysis and staining

The mice were euthanized after reaching the modeling time point, and the livers were collected and weighed for further analysis. Livers were fixed in 4% formalin followed by paraffin or optimal cutting temperature compound embedding and then sectioned transversely (5um thick). The liver sections were stained with Hematoxylin and eosin staining (HE) respectively. NAFLD activity score (NAS) following HE analysis is the sum of the scores of three components, including steatosis (0–3), lobular inflammation (0–3), and hepatocyte ballooning (0–2). Oil Red staining was performed with the frozen liver sections. The primary antibodies we used in immunofluorescence staining was listed in Supplementary Table S2. The staining process was concluded upon visual inspection under a microscope. Images were captured by Zeiss mage System or Leica system.

### 2.5 Masson’s trichrome stain

To assess and visualize fibrosis, Masson’s trichrome staining was performed. Liver tissues were fixed in 4% formalin for 24 h, then embedded in paraffin and sectioned at a thickness of 5 μm. After deparaffinization and rehydration, the sections underwent Masson’s trichrome staining (25,088, Polysciences, NY, United States) according to the manufacturer’s instructions and established protocols described in previous studies.

### 2.6 Real time quantitative PCR

Total RNA was extracted using RNA isolater Total RNA Extraction Reagent (R401-01, Vazyme, Nanjing, China) and quantified using a NanoDrop 6,000 spectrophotometer. Reverse transcription was performed using HiScript III RT SuperMix for qPCR (+gDNA wiper) (R323-01, Vazyme) and ChamQ Universal SYBR qPCR Master Mix (Q711-02, Vazyme) on 7,500 or Q6 Fast Real time PCR system. β-actin was used as the internal control for mRNA quantification. Primer sequences are listed in Supplementary Table S1.

### 2.7 Western blotting analysis

Proteins were extracted from cells and tissues were harvested and lysed in lysate buffer (PC101, Epizyme, Shanghai, China) with protease inhibitor (no.04693116001, Roche, Basel, Swiss) and phosphatase inhibitor (GRF102, Epizyme). Protein concentrations were measured using the BCA kit (ZJ103, Epizyme). Equal amounts of denatured proteins were incubated at 100 C for 10  min, separated by SDS-PAGE, and transferred onto PVDF membranes (IPVH00010, Millipore, Boston, Massachusetts, United States). Membranes were incubated with primary antibodies (list in Supplementary Table S2), followed by incubation with secondary antibodies and treatment with the chemiluminescence kit (34,577, Thermo Fisher Scientific, Waltham, Massachusetts, United States) and scanned with a gel imaging analysis system (Tanon 4,100, Tanon Science and Technology, Shanghai, China), and semi-quantitative analyzed by ImageJ (version1.52i).

### 2.8 Oil red O staining

After being treated with FFA for 48 h, the medium of cell was removed and then the cells were fixed with 4% paraformaldehyde for 15 min. Next, the diluted oil red O (0.6% oil red O in isopropanol: H2O = 3:2) was added to the cell for 20 min. After being washed with phosphate buffered saline (PBS) three times, an optical microscope was employed to observe the formation of lipid droplets. Each experiment was performed in triplicate. Subsequently, a standardized quantitative analysis of Oil Red O staining (calculated as the ratio of Oil Red O-positive area to total cell area) was performed using ImageJ.

### 2.9 Determination of intracellular TG content

To investigate the intracellular content of TG, cells were washed with PBS, harvested by trypsinization, and then resuspended in PBS. Subsequently, the cell suspension was homogenized by sonication for 5 min. Triglyceride content was determined using a commercial TG assay kit (A110-1-1, Nanjing jiancheng Biotechnology) according to the manufacturer’s protocol. The protein concentration was determined by the BCA protein assay kit, and then the intracellular content of TG was normalized to the total protein concentration in the cell lysates.

### 2.10 Proteomic analysis

Total protein extracted from mouse liver tissues was digested with trypsin. The resulting peptides were desalted using SOLA™ SPE 96-well plates. Prior to LC-MS/MS analysis, each sample was spiked with iRT reagent (Biognosys, Thermo Fisher Scientific) at a 1:20 volume ratio as an internal standard. All mass spectrometry raw data were merged and processed using DIA-NN software for database search and quantitative analysis in data-independent acquisition (DIA) mode. Significantly differentially expressed proteins were subjected to functional annotation using GO term and KEGG pathway enrichment analyses. Proteomic profiling and data processing were conducted by Shanghai Luming Biological Technology Co., Ltd. (Shanghai, China).

### 2.11 Immunofluorescence staining

Immunofluorescence staining analysis was carried out in 4 μm-thick formalin-fixed and paraffin-embedded mouse liver samples. The liver sections were blocked in 3% BSA in 0.3% Triton X-100 in PBS buffer for 1  h, then incubated overnight at 4 °C with indicated primary antibodies. Corresponding secondary antibodies (A11008, A-11005, Invitrogen) were applied for 1 h at room temperature. DAPI (C1005, Beyotime) was used for nuclear staining. The samples were observed and imaged using a fluorescent microscope (DMi8 thunder, Leica).

### 2.12 CO-immunoprecipitation (CO-IP)

Cell line was lysed using NP-40 lysis buffer (P0013F, Beyotime, Shanghai, China) at 4 °C, followed by centrifugation 12,000 rpm for 20 min. The cell lysates were then collected and incubated with Protein A/G PLUS-Agarose beads (sc-2003, Santa Cruz Biotechnology, CA, United States) at room temperature with mixing for 1–2 h, and were subsequently co-incubated with the indicated antibodies at 4 °C overnight. Next, the protein was eluted from beads with SDS buffer at 100 °C for 10 min. Immunoblotting was performed on the immune complex using the indicated primary antibodies, followed by detection with their respective secondary antibodies.

### 2.13 Measurement of lactate

To determine the concentration of lactate levels in cells, cells were lysed with buffer on ice, then centrifuged at 12,000 rpm for 15 min at 4 °C to isolate the supernatant. The lactate content in the supernatant was subsequently quantified utilizing an L-Lactate Assay Kit (MAK329, Sigma-Aldrich), adhering strictly to the protocol outlined by the kit’s manufacturer.

### 2.14 Statistical analysis

Quantitative values of data were expressed as mean ± standard error of the mean (SEM).

Student’s two-tailed t-test was performed to compare the means of two-group samples, Statistical differences among multiple groups were analyzed by one-way ANOVA followed by Brown-Forsythe test (for data showing homogeneity of variance). The non-parametric Mann-Whitney U test was used for statistical analysis in ordinal data. GraphPad Prism Software (Version 9.5.0; Graph Pad Software, Inc., San Diego, CA, United States) was used for the final data analysis. p value <0.05 was considered as significant. Randomization and blinding manners were used whenever possible.

## 3 Results

### 3.1 EMMPRIN knockdown suppressed steatosis and inflammation *in vitro*

**FIGURE 1:**
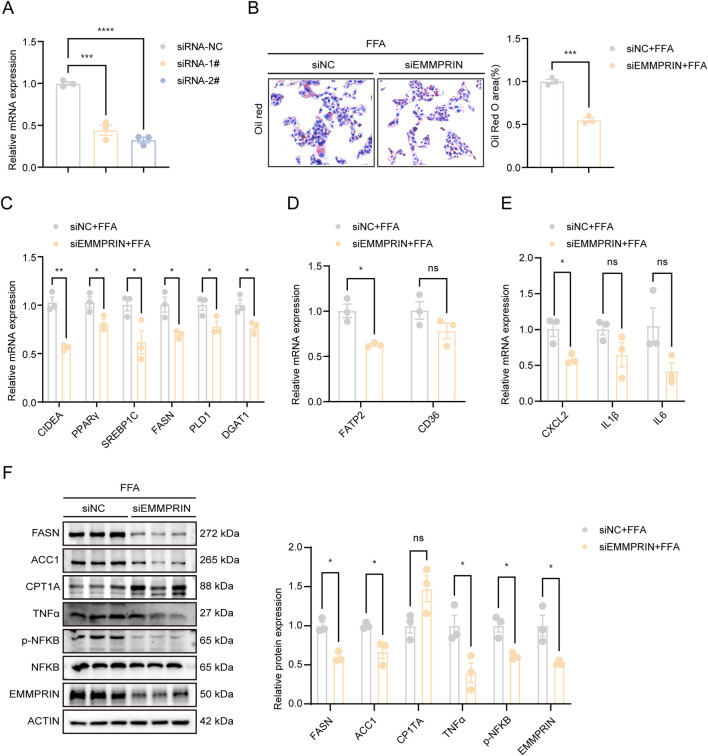
EMMPRIN knockdown suppressed steatosis and inflammation *in vitro*. **(A)** The EMMPRIN expression by qPCR analysis in THLE-2 cells treated with siRNA of EMMPRIN (n = 3/group). **(B)** Representative Oil Red O staining and quantification of THLE-2 cells in the indicated groups stimulated with FFA for 48h. **(C–E)** The gene expression of fatty acid synthesis **(C)** and uptake **(D)**, and inflammations **(E)** by qPCR analysis in cells. **(F)** Western blot analysis and quantification of protein levels in cells. Data are presented as mean ± SEM. (*p < 0.05, **p < 0.01, ***p < 0.001; ****p < 0.0001; ns, not significant).

To assess the impact of EMMPRIN on MASLD *in vitro*, we first established a cellular model of steatosis by treating THLE-2 cells with a mixture of free fatty acids (FFA). A dose- and time-response analysis confirmed that treatment with 1.0 mM FFA for 48 h, which induced significant lipid accumulation (Supplementary Figure S1B) and EMMPRIN upregulation (Supplementary Figures S1C–E), maintained a high cell viability of 86.3% (Supplementary Figure S1A), ensuring that the subsequent observations reflected specific lipid overload effects. Upon this optimized condition, we knocked down EMMPRIN expression using siRNA, which resulted in a significant reduction of EMMPRIN levels (Figure 1A). Subsequent Oil Red O staining and TG concentration assessment demonstrated a significant reduction in lipid content upon EMMPRIN suppression, as compared to the control group (Figure 1B; Supplementary Figure S1F). Furthermore, the expression of genes involved in lipogenesis and lipid transport was significantly downregulated, while oxidation-related markers were upregulated following EMMPRIN knockdown (Figures 1C,D,F). Additionally, the inflammatory response was alleviated by EMMPRIN knockdown (Figures 1E,F). To examine potential proliferative effects, we assessed proliferation markers PCNA and MCM2 after EMMPRIN knockdown. Results showed no significant changes in their expression levels (Supplementary Figure S6D), indicating that EMMPRIN suppression did not induce proliferative alterations. Collectively, these findings suggested that EMMPRIN knockdown mitigated steatosis and inflammation *in vitro*, supporting a pro-steatotic role of EMMPRIN in MASLD.

### 3.2 Overexpression of EMMPRIN exacerbated experimentally induced fatty liver disease in mice

**FIGURE 2:**
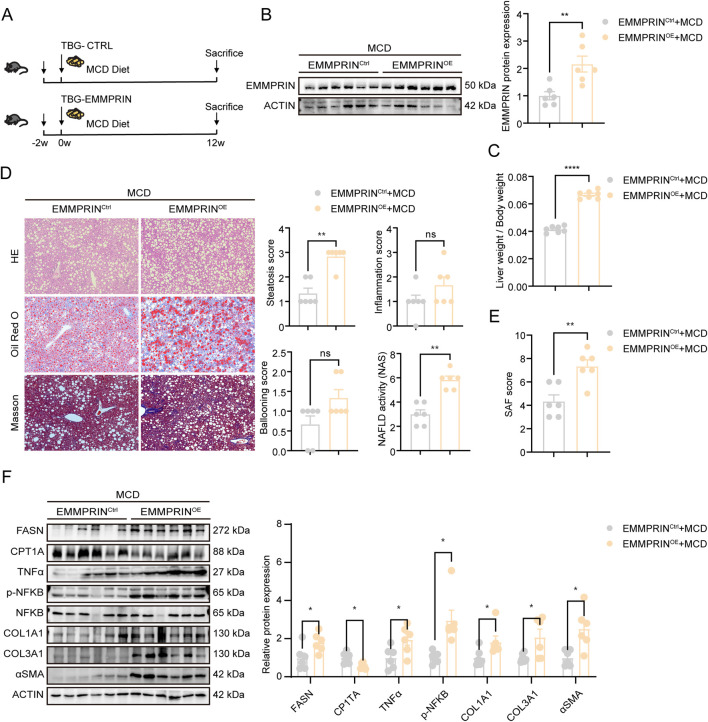
Overexpression of EMMPRIN exacerbated experimentally induced fatty liver disease in mice. **(A)** Schematic diagram of MCD diet-induced MASH models in EMMPRIN^OE^ or EMMPRIN^Ctrl^ mice. **(B)** The EMMPRIN expression in liver by Western blot analysis inEMMPRIN^OE^ and EMMPRIN^Ctrl^ mice (n = 6/group). **(C–E)** Liver weight to body weight **(C)**, HE staining, Oil Red O staining and Masson staining of liver and quantification of the NAS score **(D)**, quantification of the SAF score **(E)** from EMMPRIN^OE^ and EMMPRIN^Ctrl^ mice. **(F)** Western blot analysis and quantification of protein levels in liver in the indicated groups. Data are presented as mean ± SEM. (*p < 0.05, **p < 0.01, ***p < 0.001; ****p < 0.0001; ns, not significant).

To investigate the role of EMMPRIN in the development and progression of metabolic-associated steatohepatitis, a choline-deficient diet model was employed. The MCD diet induces steatohepatitis histologically similar to human NASH, and further promotes oxidative stress, enhances inflammation and fibrosis, and raises systemic lipid concentrations (Farrell et al., 2019; Leclercq et al., 2000). To investigate the impact of EMMPRIN on the MASH phenotype, we achieved hepatic overexpression of EMMPRIN via tail vein injection of an AAV8 vector carrying the EMMPRIN gene (Figures 2A,B). Subsequently, MASH was induced in the mice. EMMPRIN overexpression resulted in an increased liver-to-body weight ratio in MCD-fed mice (Figure 2C). Histological assessments using H&E and Oil Red O staining revealed greater lipid droplet accumulation in the EMMPRIN-overexpressing group, accordingly, the NAFLD activity score was higher in these mice compared with controls (Figure 2D). Masson’s trichrome staining and the SAF scoring system were applied to assess liver fibrosis. The results revealed increased collagen deposition and a higher fibrosis SAF score following EMMPRIN overexpression (Figures 2D,E). At the molecular level, EMMPRIN overexpression upregulated the expression of lipogenic gene FASN, the inflammatory signaling pathway NFKB (TNFα and p-NFKB), and fibrotic markers including α-SMA, COL1A1, and COL3A1, while downregulating the expression of CPT1A, a key gene that promotes fatty acid oxidation (Figure 2F). Furthermore, to evaluate whether EMMPRIN overexpression induced hepatocyte proliferation and potential cancer phenotypes, we examined the expression of proliferation markers PCNA, MCM2. Western blot analysis showed no significant difference in the levels of these proliferation markers between the EMMPRIN-overexpressing group and the control group (Supplementary Figure S6A). Further immunofluorescence assessment of Ki67 expression in albumin-positive hepatocytes in liver tissues also revealed no difference in the Ki67-positive hepatocytes between the two groups (Supplementary Figure S6B), indicating that no cancer-related phenotype was observed under the experimental conditions of this study. Taken together, these findings demonstrated that EMMPRIN overexpression aggravated MCD diet-induced fatty liver disease in mice.

### 3.3 Hepatocyte-specific EMMPRIN loss attenuated histological progression of MASH

**FIGURE 3:**
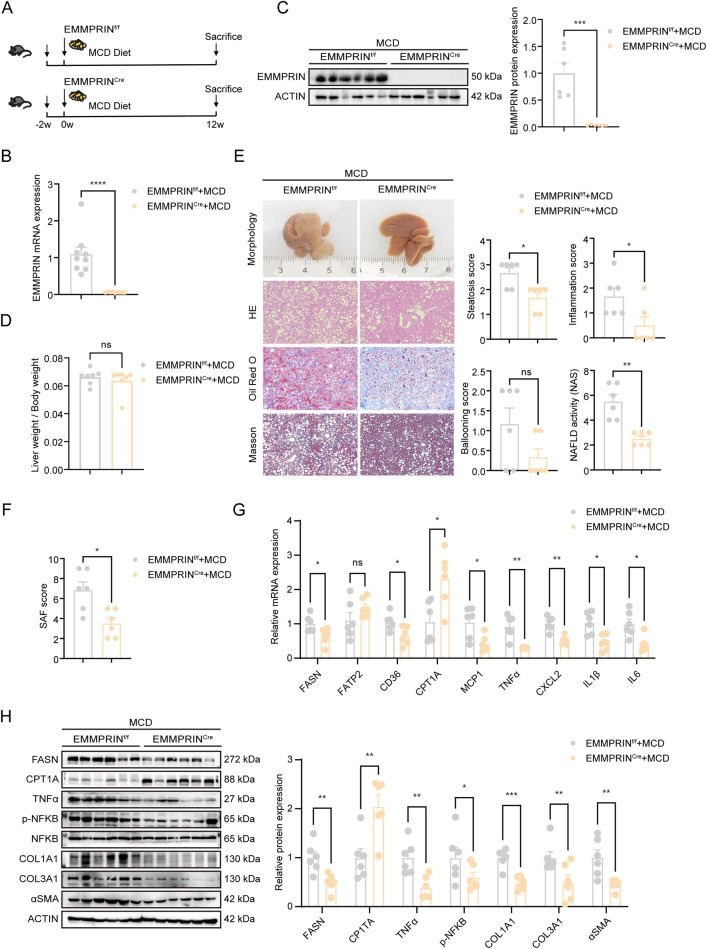
Hepatocyte-specific EMMPRIN loss attenuated histological progression of MASH. **(A)** Schematic diagram of MCD diet-induced MASH models in EMMPRIN^f/f^ and EMMPRIN^Cre^ mice. **(B,C)** The EMMPRIN expression in liver by qPCR and Western blot analysis in EMMPRIN^f/f^ and EMMPRIN^Cre^ mice (n = 6/group). **(D–F)** Liver weight to body weight **(D)**, HE staining, Oil Red O staining and Masson staining of liver and quantification of the NAS score **(E)**, quantification of the SAF score **(F)** from EMMPRIN^f/f^ and EMMPRIN^Cre^ mice. **(G)** The gene expression by qPCR analysis in the indicated groups. **(H)** Western blot analysis and quantification of protein levels from liver in the indicated groups. Data are presented as mean ± SEM. (*p < 0.05, **p < 0.01, ***p < 0.001; ****p < 0.0001; ns, not significant).

To investigate the physiological role of EMMPRIN under pathogenic stress by inducing MASH in mice. Hepatocytic EMMPRIN-deficient mice (EMMPRIN^Cre^) were generated via CRISPR/Cas9-mediated gene editing (Figure 3A). Both EMMPRIN mRNA and protein levels were significantly reduced in the livers of EMMPRIN^Cre^ group (Figures 3B,C), although the liver-to-body weight ratio was unchanged (Figure 3D). Notably, EMMPRIN deletion ameliorated the pathological morphology of liver tissues in MASH mice (Figure 3E). Histological evaluation showed marked reductions in key MASH features—including steatosis and lobular inflammation—which collectively led to a lower NAFLD activity score (Figure 3E). The fibrosis components of Masson trichrome staining and SAF score showed that the degree of fibrosis was reduced in the EMMPRIN^Cre^ group (Figures 3E,F). Expression spectrum analysis further demonstrated that EMMPRIN deficiency led to the suppression of key genes involved in fatty acid synthesis, uptake, inflammation, and fibrogenesis (Figures 3G,H). This was also accompanied by a notable upregulation of fatty acid oxidation, these changes may co-contribute to the reduced severity of MASH observed in these animals (Figures 3G,H). In addition, to exclude the potential effect of EMMPRIN deletion on hepatocyte proliferation, we assessed proliferation markers and found no significant difference between the EMMPRIN-knockout and control groups (Supplementary Figure S6C). This further supports that the role of EMMPRIN in MASH is primarily mediated through metabolic regulation. Taken together, these data suggested that conditional EMMPRIN knockout in hepatocytes provided protection against the progression of MASH, as supported by both histopathological and molecular evidence.

### 3.4 EMMPRIN deletion remodeled the proteome to alleviate MASH

**FIGURE 4:**
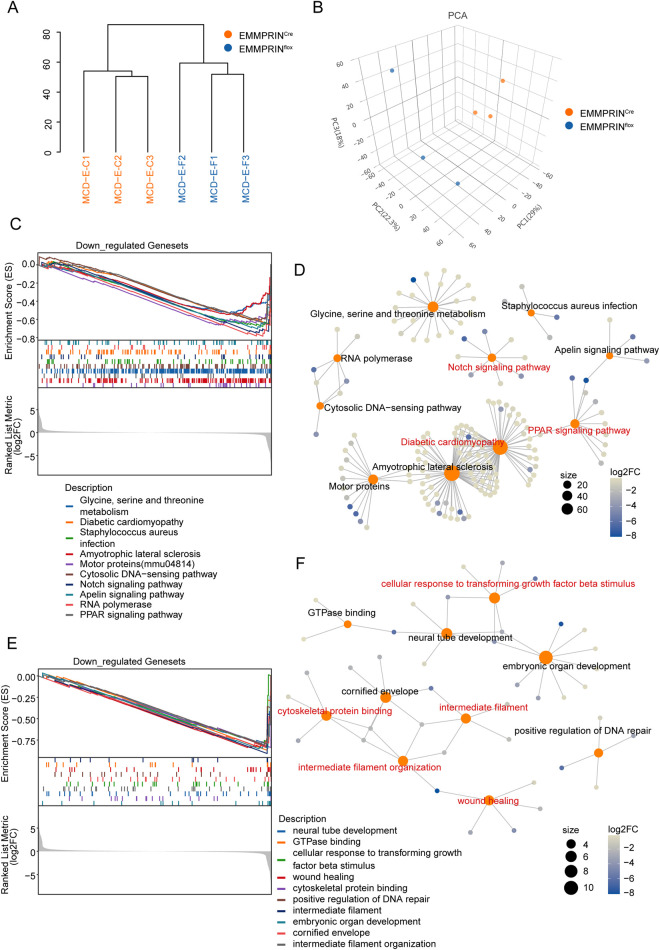
EMMPRIN deletion remodeled the proteome to alleviate MASH. **(A)** The samples tree plot of proteomic sequencing in EMMPRIN^f/f^ and EMMPRIN^Cre^ mice induced by MCD diet groups (n = 3/group). **(B)** Sample distribution profiles of EMMPRIN^f/f^ and EMMPRIN^Cre^ mice groups analyzed by PCA. **(C,D)** Combined GSEA plot **(C)** and pathway network plot **(D)** of the top 10 signaling pathway in KEGG based on proteomic-seq data. **(E,F)** Combined GSEA plot **(E)** and pathway network plot **(F)** of the top 10 signaling pathway in GO based on proteomic-seq data.

To elucidate the underlying molecular mechanisms by which EMMPRIN deficiency exerts protective effects in MASH, we performed quantitative proteomic profiling using Data-Independent Acquisition (DIA)-based mass spectrometry on liver tissues from MASH mice with hepatocyte-specific EMMPRIN deletion (Figure 4A). Principal component analysis (PCA) showed a clear separation between EMMPRIN^Cre^ and EMMPRIN^f/f^ groups, thus demonstrating substantial proteomic alterations upon EMMPRIN loss (Figure 4B). We further conducted Kyoto Encyclopedia of Genes and Genomes (KEGG) pathway enrichment as well as Gene Ontology (GO) functional analyses using GSEA to identify biological processes modulated by EMMPRIN deficiency (Figures 4C,E). Proteomic analysis of the data identified the top ten enriched pathways, among which EMMPRIN^Cre^ mice exhibited significant downregulation in pathways related to lipid metabolism and inflammatory responses—including the PPAR signaling pathway, diabetic cardiomyopathy, and Notch signaling—as supported by both KEGG analysis (Figure 4D) and gene expression heatmaps (Supplementary Figures S2A,B). GSEA also indicated suppression of fibrotic processes in EMMPRIN^Cre^ mice, such as cellular response to TGF-β stimulus, wound healing, intermediate filament organization, and cytoskeletal protein binding, which was consistent with GO term enrichment (Figure 4F) and corresponding heatmap visualization (Supplementary Figure S2C). Collectively, these results demonstrated that EMMPRIN deficiency attenuated MASH progression through coordinated downregulation of pathways that are central to lipid metabolism, inflammation, and fibrosis.

### 3.5 EMMPRIN formed a functional complex with MCT1 to disrupt metabolic homeostasis

**FIGURE 5:**
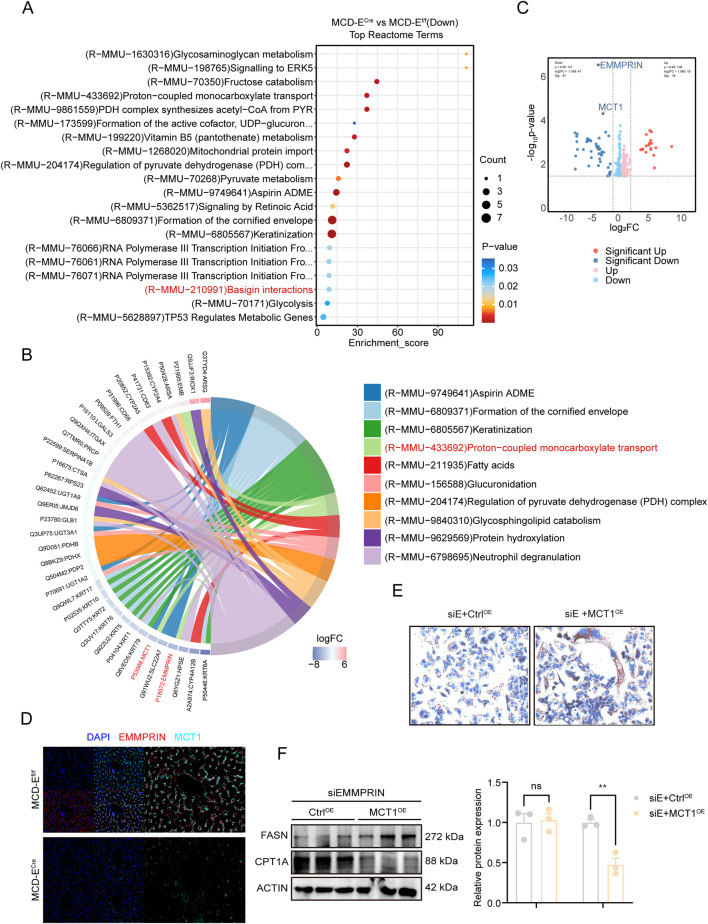
EMMPRIN formed a functional complex with MCT1 to disrupt metabolic homeostasis. **(A)** The Reactome enrichment analysis of differentially proteins from proteomic sequencing in EMMPRIN^f/f^ and EMMPRIN^Cre^ mice induced by MCD diet groups. **(B)** Cirplot showed pathways enrichment analysis of regulated “Basigin interactions” in EMMPRIN deletion mice fed the MCD diet compared to controls. **(C)** Volcano plot of proteomic-seq data. **(D)** MCT1 and EMMPRIN double staining in liver of mice in the indicated groups. **(E,F)** Representative Oil Red O staining **(E)** and Western blot analysis **(F)** in THLE-2 cells treated with MCT1^OE^ or Control under EMMPRIN knockdown. Data are presented as mean ± SEM. (*p < 0.05, **p < 0.01, ***p < 0.001; ****p < 0.0001; ns, not significant).

**FIGURE 6:**
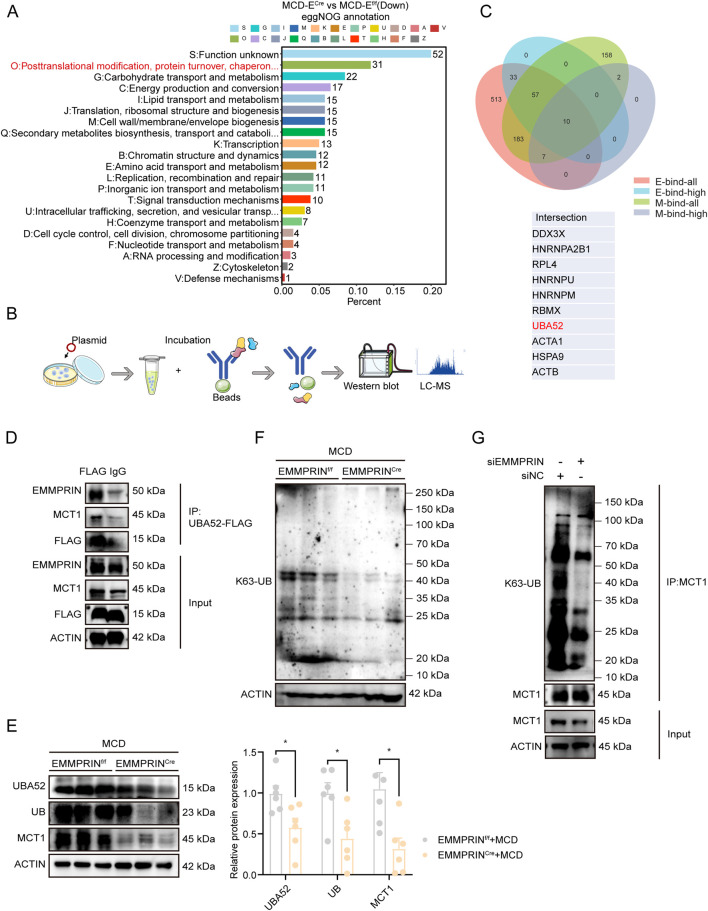
UBA52-mediated K63-linked ubiquitination of MCT1 promoted downstream of EMMPRIN. **(A)** The eggNOG enrichment analysis based on proteomic-seq data from proteomic sequencing in EMMPRIN^f/f^ and EMMPRIN^Cre^ mice induced by MCD diet groups. **(B)** Schematic diagram of mass spectrometry and co-immunoprecipitation. **(C)** Venn diagram displaying proteins that co-interact with both HA-EMMPRIN and FLAG-MCT1 by mass spectrometry analysis. **(D)** Reciprocal co-IP analysis of EMMPRIN, UBA52 and MCT1 in THLE-2 cells. IgG was used as a negative control. **(E)** Western blot analysis and quantification of protein levels from liver in the EMMPRIN^f/f^ and EMMPRIN^Cre^ mice groups. **(F)** Western blot analysis in liver and quantification of K63-UB levels in the indicated groups. **(G)** Reciprocal co-IP analysis in K63-UB levels of MCT1 in THLE-2 cells. Data are presented as mean ± SEM. (*p < 0.05, **p < 0.01, ***p < 0.001; ****p < 0.0001; ns, not significant).

Given the ameliorative impact of EMMPRIN deficiency, we next sought to identify the key mediators downstream of EMMPRIN. We focused on its interaction network—specifically “Basigin interactions” (Figure 5A). Within this context, volcano plot analysis of proteomic data revealed that EMMPRIN knockout led to significant downregulation of its chaperone, monocarboxylate transporter 1 (MCT1) (Figure 5C), suggesting its potential involvement in EMMPRIN-mediated biological processes. Further functional annotation indicated that the EMMPRIN–MCT1 complex was primarily associated with “proton-coupled monocarboxylate transport” (Figure 5B). To validate this interaction, immunofluorescence staining confirmed the co-localization of EMMPRIN and MCT1 on the plasma membrane of liver cells (Figure 5D). Consistently, at the cellular level, exogenous overexpression of HA-tagged EMMPRIN in hepatocytes followed by co-immunoprecipitation (Co-IP) assays further substantiated the direct binding between EMMPRIN and MCT1 (Supplementary Figure S3D). Moreover, we observed MCT1 protein levels were markedly reduced following EMMPRIN deletion by immunofluorescence assays and Western blot (Figures 5D, 6E), supporting a functional partnership between the two molecules. To determine whether EMMPRIN exerted its regulatory effects through MCT1, we conducted rescue experiments. Results demonstrated that overexpression of MCT1 reversed the attenuation of lipid accumulation caused by EMMPRIN knockdown, as evidenced by increased lipid droplet content (Figure 5E). Concurrently, the expression of FASN was not significantly changed, while that of CPT1A was markedly downregulated (Figure 5F). Collectively, these findings suggested that EMMPRIN may form a functional complex with MCT1, and its disruption of hepatic metabolic homeostasis largely depends on the regulation of fatty acid oxidation, thereby contributing to the pathogenesis of fatty liver disease.

### 3.6 UBA52-mediated K63-linked ubiquitination of MCT1 promoted downstream of EMMPRIN

Subsequent in-depth analysis of the proteomic network regulated by EMMPRIN revealed significant alterations in protein post-translational modifications (Figure 6A). Ubiquitination, as a highly dynamic modification, played a critical role in cellular stress responses such as lipid overload (Min et al., 2025). It contributed to the onset and progression of MASLD by affecting insulin resistance, endoplasmic reticulum stress, mitochondrial dysfunction, and lipid autophagy (Zhang et al., 2025). Notably, we observed a marked decrease in global ubiquitination levels following EMMPRIN knockout (Supplementary Figure S3A), suggesting that EMMPRIN may participate in the regulation of ubiquitination. Based on these findings, we first investigated whether EMMPRIN is involved in ubiquitination regulation by affecting autophagy. Detection of autophagy markers LC3 and p62 showed no significant changes in the LC3-II/LC3-I ratio or p62 protein levels in either EMMPRIN-knockout mouse liver tissues or *in vitro* cell models (Supplementary Figures S4A,B), indicating that EMMPRIN deletion does not affect the autophagy process. This result suggests that EMMPRIN may participate in ubiquitination regulation through autophagy-independent pathways. To further elucidate the specific mechanism, we hypothesized that the EMMPRIN/MCT1 might be subject to ubiquitin-dependent regulation. By overexpressing HA-tagged EMMPRIN and FLAG-tagged MCT1 in hepatocytes, combined with co-immunoprecipitation-mass spectrometry analysis, we identified UBA52 as a common binding partner for both proteins (Figures 6B,C). UBA52, as a ubiquitin precursor protein, plays a fundamental role in ubiquitin-mediated protein modification (Lund et al., 1985; Martínez-Férriz et al., 2022). Furthermore, we identified a positive regulatory relationship and interaction between EMMPRIN and UBA52 by co-immunoprecipitation and immunofluorescence assays (Figure 6E; Supplementary Figure S5A). Further co-immunoprecipitation confirmed that UBA52 simultaneously binds both EMMPRIN and MCT1 (Figure 6D). It has been reported that UBA52 undergoes rapid deubiquitination shortly after ribosomal synthesis, yielding free ubiquitin and ribosomal protein L40 (Martínez-Férriz et al., 2022; Tiwari et al., 2022), Furthermore, UBA52 has been shown to participate in cellular responses to oxidative damage (Cai et al., 2024; Tiwari et al., 2023). These findings prompted us to investigate whether EMMPRIN influenced ubiquitin homeostasis. Mechanistically, we found that EMMPRIN deletion led to a significant reduction in total ubiquitin levels (Figure 6E) indicating that the loss of EMMPRIN was likely to disrupt ubiquitin equilibrium. We further examined the two major types of ubiquitin chain linkages: K48-linked polyubiquitination, which primarily targets proteins for degradation, and K63-linked polyubiquitination, which controls various protein properties, including protein-protein interaction and stabilization (Rahman and Wolberger, 2024; Swatek and Komander, 2016). The results demonstrated that K63-linked polyubiquitination was significantly decreased in EMMPRIN-knockout mice (Figure 6F), whereas K48-linked modification remained unaltered (Supplementary Figure S3B). More importantly, K63-linked ubiquitination of MCT1 was markedly reduced upon EMMPRIN knockdown, indicating impaired protein stability of MCT1 (Figure 6G).

Notably, our previous research has confirmed that the functional complex formed by EMMPRIN and MCT1 participates in proton-coupled monocarboxylate transport (Figure 5B), which is critical for lactate shuttling. Based on this, to systematically analyze the biological function of the EMMPRIN-UBA52-MCT1 signaling axis, we further investigated the effect of EMMPRIN knockdown on lactate metabolism. Experimental results showed that intracellular lactate levels were significantly increased in the EMMPRIN-knockdown hepatocyte compared with controls (Supplementary Figure S5B). This phenomenon corroborates the aforementioned molecular events of decreased K63 ubiquitination and reduced MCT1 stability, further confirming the impairment of lactate transport. More importantly, we also found that global protein lactylation levels were elevated in the livers of EMMPRIN-knockout mice (Supplementary Figure S3C), suggesting that accumulated intracellular lactate may function as a signaling molecule through protein lactylation modification (Zhang et al., 2019; Chen et al., 2021), thereby regulating downstream inflammatory and fibrotic responses. These results collectively reveal that EMMPRIN may regulate lactate metabolic balance by affecting MCT1 stability through UBA52-mediated K63-linked ubiquitination. However, the complex underlying mechanisms require further exploration in future studies.

## 4 Discussion

This study demonstrates that EMMPRIN played a critical role in the progression of metabolic-associated steatohepatitis (MASH) through the ubiquitination of MCT1. Under MCD diet induction, EMMPRIN-overexpressing mice developed exacerbated hepatic steatosis, inflammatory responses, and fibrosis, whereas hepatocyte-specific EMMPRIN knockout conferred significant protection against these pathological features. Using an integrated approach combining proteomics, mass spectrometry, co-immunoprecipitation, and Western blot analyses, we identified MCT1 and the ubiquitin precursor UBA52 as direct targets of EMMPRIN. Mechanistically, EMMPRIN downregulated UBA52 expression, thereby reducing the intracellular free ubiquitin pool and subsequently impairing K63-linked polyubiquitination of MCT1, which compromised its stability and function. Moreover, EMMPRIN deficiency induced a global increase in protein lactylation, likely resulting from dysregulated lactate metabolism secondary to impaired MCT1 -mediated lactate shuttle. These findings establish a novel regulatory axis wherein EMMPRIN governs MASH progression through ubiquitin-dependent control of MCT1, positioning EMMPRIN as a promising therapeutic target for MASH intervention.

In genetically susceptible individuals, nutrient overload and insulin resistance initiate hepatic triglyceride accumulation, establishing the foundation for MASLD (Huang et al., 2025). Excess production of reactive oxidative metabolites from lipids, free fatty acid oxidation, cytokine-mediated inflammation, apoptosis, and necrosis collectively contribute to the recruitment and activation of immune cells and hepatic stellate cells (Huang et al., 2025; Sharma et al., 2023). The convergence of these pathological processes—such as sustained inflammatory responses and progressive fibrogenesis—disrupted normal liver architecture and function, driving the progression of MASLD. In MCD diet-induced MASH models, persistent lipid overload, chronic liver injury, inflammatory responses, and HSC activation were considered central mechanisms of disease progression (Farrell et al., 2019; Rinella and Green, 2004). Consequently, reversing these processes has become a crucial strategy in current anti-MASH therapy. In this study, EMMPRIN knockout significantly alleviated hepatic lipid accumulation, as evidenced by downregulation of lipid synthesis-related genes and upregulation of the fatty acid oxidation. These alterations in lipid metabolism pathways closely aligned with the histological attenuation of steatosis. Lipid overload–derived reactive oxygen metabolites and free fatty acids activated the NF-κB signaling pathway, promoting the expression of pro-inflammatory cytokines such as TNFα, IL-1β, and IL-6 (Saadati et al., 2025). Our findings also demonstrated that EMMPRIN overexpression further aggravated this process, fostering a state of chronic inflammation, while EMMPRIN knockout exerted the opposite effect. Persistent inflammatory responses activated hepatic stellate cells, stimulating the synthesis and deposition of extracellular matrix components, including collagens (COL1A1, COL3A1), ultimately leading to liver fibrosis. These findings are consistent with the fibrotic phenotype observed in our study. In the present work, EMMPRIN knockout mitigated hepatic lipid accumulation, inflammatory injury, and fibrotic progression. Proteomic enrichment analysis provided a molecular basis for these phenotypic changes, revealing significant modulation of pathways such as PPAR signaling, diabetic cardiomyopathy–related signaling, and Notch signaling upon EMMPRIN deletion. Moreover, EMMPRIN deficiency impaired cellular responses to TGF-β and wound-healing processes. The regulation of these pathways corresponded with the hepatoprotective effects observed and may underlie the beneficial outcomes; however, the precise functional contribution of each pathway warrants further investigation. Additionally, neither EMMPRIN overexpression nor knockout induced significant changes in the expression of proliferation markers PCNA, MCM2, and Ki67, thereby excluding the possibility that EMMPRIN participates in MASH progression via modulation of cell proliferation or induction of tumor-like phenotypes. Collectively, our results suggest that targeting EMMPRIN is a promising strategy for MASH treatment, potentially through the regulation of these pathways.

Our findings establish that MCT1 as a molecular chaperone of EMMPRIN, mediates the latter’s regulatory role in the development and progression of MASH. Monocarboxylate transporters (MCTs), which belong to the solute carrier 16 (SLC16) family, facilitate the transmembrane transport of short-chain monocarboxylates, hormones, nutrients, and amino acids. MCTs share common substrates including lactate, pyruvate, ketone bodies, and short-chain fatty acids, facilitating their transport across the plasma membrane (Felmlee et al., 2020; Halestrap, 2012; Singh et al., 2023). Notably, MCT1 has been widely characterized for its critical role in lactate shuttling in cancer cells (Chen et al., 2021; Park et al., 2018). Previous reports have shown that MCT1 deficiency in adipose tissue exacerbates local inflammation and promotes high-fat diet -induced insulin resistance in mice (Lin et al., 2022). Furthermore, liver-specific knockout of MCT1 reduces food-anticipatory activity in mice (Martini et al., 2021), underscoring its important role in energy metabolism regulation. Importantly, the present study reveals a previously unrecognized mechanism whereby ubiquitination of MCT1 serves as a critical event in EMMPRIN-dependent regulation of MASH, expanding the functional spectrum of MCT1 in metabolic disease.

UBA52, a ubiquitin-ribosomal fusion protein that simultaneously serves as a ubiquitin precursor and a putative E3 ligase, was regulated by EMMPRIN in the context of MASH. EMMPRIN loss lowered the total ubiquitin pool, which selectively reduced K63-linked polyubiquitination of MCT1 without altering K48-linked chains. The ubiquitin molecule contains seven lysine (Lys) residues that can form various types of polyubiquitin chains through iterative processes. Among these, Lys48 (K48)- and Lys63(K63)-linked chains remained the most extensively characterized to date, with the latter playing a particularly important role in non-degradative signaling (Hicke et al., 2005). Ubiquitination directly influenced the stability, activity, and protein interactions of target molecules, making it crucial for understanding ubiquitin-dependent cellular processes. The decreased K63 signal was accompanied by lower MCT1 abundance and impaired lactate export, ultimately leading to global protein lactylation and attenuated MASH progression. To exclude the possibility that EMMPRIN modulates ubiquitination indirectly through autophagy, we monitored LC3-II/I conversion and p62 levels; neither marker was affected by EMMPRIN deletion *in vivo* or *in vitro*. Thus, the EMMPRIN-UBA52 axis appears to act independently of the autophagic machinery. Future work will extend to explore whether EMMPRIN participates in the regulation of the ubiquitin‒proteasome system via alternative mechanisms, such as modulating the activity of deubiquitinating enzymes (DUBs), or interacting with other E3 ubiquitin ligases. Such multidimensional analyses will provide a more complete picture of how EMMPRIN governs protein stability and metabolic reprogramming in MASH.

In summary, this study systematically delineates the role of the EMMPRIN-MCT1 complex and its regulation by UBA52-mediated K63-linked ubiquitination in MASH pathogenesis at the molecular, cellular, and animal levels. Notably, we also identified a novel mechanistic link between EMMPRIN/MCT1 axis dysfunction and lactylation-driven metabolic reprogramming, providing a rational basis for developing EMMPRIN-targeted therapeutic strategies.

However, this study has several limitations. First, although global hyperlactylation was observed in EMMPRIN-knockout mice, the specific lactylated targets and their functional roles in MASH progression remain unclear; future studies should integrate lactylation proteomics profiling with functional validation to identify key lactylated proteins. Second, although genetic loss-of-function demonstrates EMMPRIN’s necessity, the efficacy of clinically relevant pharmacological inhibitors has not been tested in MASH models, and the therapeutic effect of EMMPRIN inhibitors needs to be evaluated in the future. Thirdly, the functional rescue experiments of UBA52 gene mice at the animal level have not yet been conducted, and further verification of its functional role in MASH is needed. Addressing these issues will help translate these findings into clinically applicable strategies.

## 5 Conclusion

This study demonstrated that EMMPRIN promoted MASH through UBA52-mediated K63-linked ubiquitination and stabilization of MCT1, ultimately influencing lactylation-dependent metabolic reprogramming. These findings establish the EMMPRIN–UBA52–MCT1 regulatory axis as a critical mechanism underlying MASH progression and highlight EMMPRIN as a promising therapeutic target.

## Data Availability

The original contributions presented in the study are included in the article/Supplementary Material, further inquiries can be directed to the corresponding authors.
