# Supplementary figures and images for: EMMPRIN deficiency alleviated metabolic-associated steatohepatitis progression via regulation of the UBA52–MCT1 axis
Source: Front Pharmacol. 2026 Jan 23;17:1706859. doi: 10.3389/fphar.2026.1706859 (PMC12875904; doi:10.3389/fphar.2026.1706859)

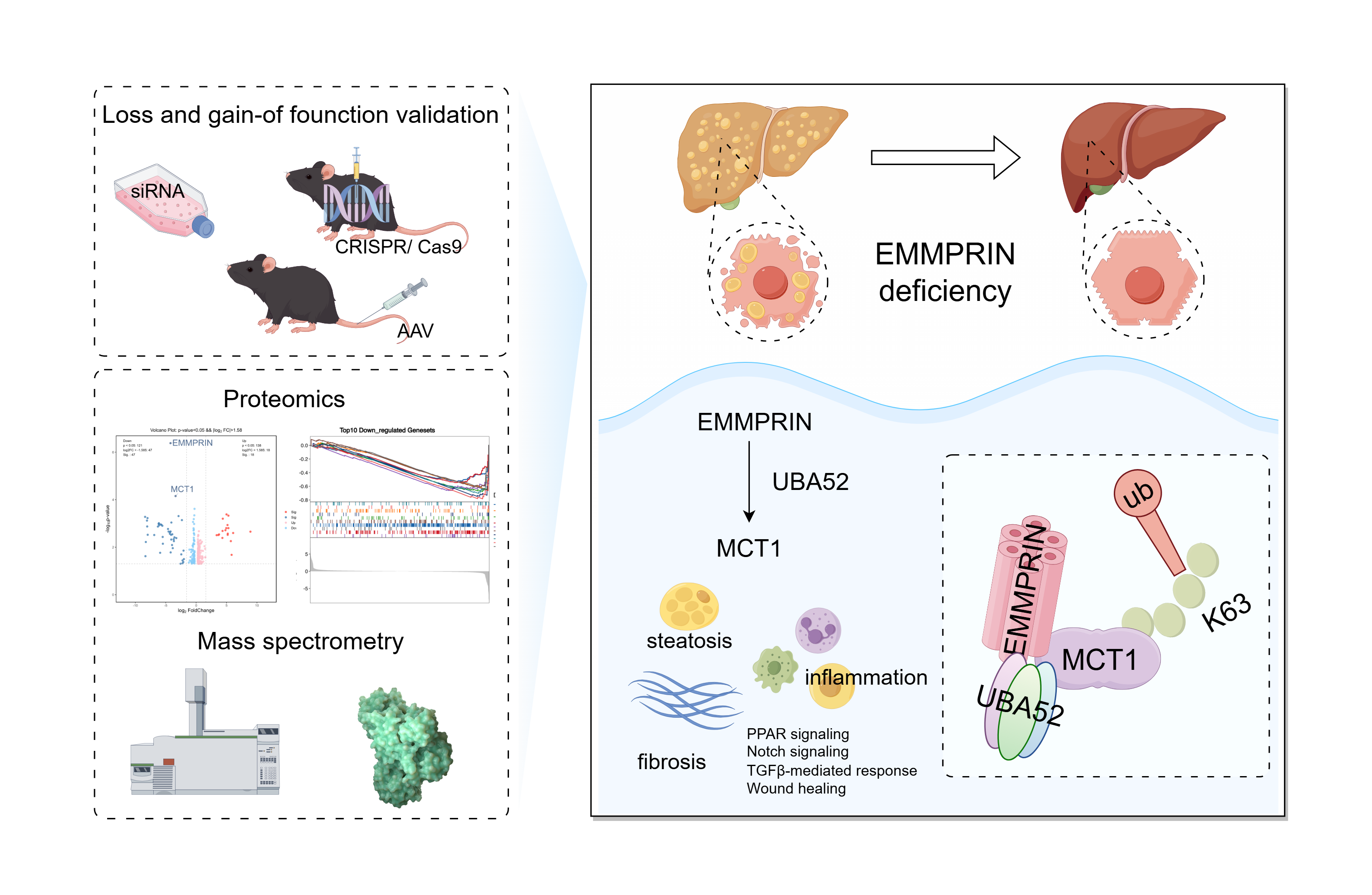

Supplement: Supplementary file 1 [file Image1.tiff]
