# Supplementary material for: EMMPRIN deficiency alleviated metabolic-associated steatohepatitis progression via regulation of the UBA52–MCT1 axis
Source: Front Pharmacol. 2026 Jan 23;17:1706859. doi: 10.3389/fphar.2026.1706859 (PMC12875904; doi:10.3389/fphar.2026.1706859)
Supplement: Supplementary file 2 [file Supplementaryfile1.docx]

**Supplementary information**

**EMMPRIN deficiency alleviated metabolic-associated steatohepatitis progression via regulation of the UBA52–MCT1 Axis**

Linying Lai^1^, Baoyuan Huang^1^, Ziping Song^1^, Xinyan Zhu^2*^, Wenzhuo Yang^1*^

**Supplementary Figures**

**Fig. S1 EMMPRIN upregulation and cell viability in a lipid overload-induced hepatocyte steatosis model**

(A) THLE-2 cells were treated with 0, 0.5, 1.0, 1.5 mM FFA for 0, 24, 48, 72 h. Cell viability was determined by CCK8(n=3). (B) The intracellular TG were stained by TG assay kit and quantified by measuring absorbance at 500 nm (n=3). (C-E) Western blot analysis and quantification of protein levels in THLE-2 cells in the indicated groups. (F) The intracellular TG was determined by TG assay kit and quantified in the indicated groups (n=3). Data are presented as mean ± SEM. (*p <0.05, **p <0.01, ***p <0.001; ****p <0.0001; ns, not significant).

**Fig. S2 EMMPRIN deletion remodeled the proteome to alleviate MASH**

(A) Heatmap illustrating the prospective target genes of EMMPRIN associated with PPAR signaling and diabetic cardiomyopathy based on proteomic-seq data (n = 3/group). (B) Heatmap illustrating the prospective target genes of EMMPRIN associated with Notch signaling. (C) Heatmap illustrating the prospective target genes of EMMPRIN associated with Cellular response to TGF-β stimulus, wound healing, intermediate filament organization, intermediate filament and cytoskeletal protein binding signaling.

**Fig. S3 UBA52-mediated K63-linked ubiquitination of MCT1 promoted downstream of EMMPRIN**

(A) The biological process enrichment analysis based on proteomic-seq data. (B-C) Western blot analysis and quantification of K48-UB and pan-lac levels in the liver from EMMPRIN^f/f^ and EMMPRIN^Cre^ micegroups. (D) Reciprocal co-IP analysis of EMMPRIN and MCT1 in THLE-2 cells.

**Fig. S4 EMMPRIN deletion does not affect hepatocyte autophagy activity**

(A) Western blot analysis and quantification of protein levels in the liver from EMMPRIN^f/f^ and EMMPRIN^Cre^ mice groups. (B) Western blot analysis and quantification of protein levels in the indicated cells. Data are presented as mean ± SEM. (*p <0.05, **p <0.01, ***p <0.001; ****p <0.0001; ns, not significant).

**Fig. S5 Validation of EMMPRIN-UBA52 interaction and analysis of lactate**

(A) UBA52 and EMMPRIN double staining in liver of mice from EMMPRIN^f/f^ and EMMPRIN^Cre^ mice groups. (B) The intracellular lactate was determined by L-Lactate Assay Kit and quantified in the indicated groups (n=6). Data are presented as mean ± SEM. (*p <0.05, **p <0.01, ***p <0.001; ****p <0.0001; ns, not significant).

**Fig. S6 EMMPRIN alteration does not significantly affect hepatocyte proliferation or tumor-related phenotypes**

(A) Western blot analysis and quantification of protein levels in the liver from EMMPRIN^Ctrl^ and EMMPRIN^OE^ mice groups. (B) Ki67 and Albumin double staining in liver of mice from EMMPRIN^Ctrl^ and EMMPRIN^OE^ mice groups. (C) Western blot analysis and quantification of protein levels in the liver from EMMPRIN^f/f^ and EMMPRIN^Cre^ mice groups. (D) Western blot analysis and quantification of protein levels in the indicated cells. Data are presented as mean ± SEM. (*p <0.05, **p <0.01, ***p <0.001; ****p <0.0001; ns, not significant).

**Supplementary Table 1. Primers for real time quantitative PCR (qPCR)**

| Primers | Sequence 5’→3’ | Supplier |
| --- | --- | --- |
| (Mouse) EMMPRIN | F: GTGGCGTTGACATCGTTGG | Sangon Biotech |
|  | R: CTATGTACTTCGTATGCAGGTCG | Sangon Biotech |
| (Mouse) FASN | F: ACCACCAGAGACCGTTATGC | Sangon Biotech |
|  | R: TGGGTTCTAGCCAGCAGAGT | Sangon Biotech |
| (Mouse) FATP2 | F: TTCAACAGCGGAGACCTCCTGA | Sangon Biotech |
|  | R: CCACGATGTCAGCGACTTCTGT | Sangon Biotech |
| (Mouse) CD36 | F: ATGGGCTGTGATCGGAACTG | Sangon Biotech |
|  | R: GTCTTCCCAATAAGCATGTCTCC | Sangon Biotech |
| (Mouse) CPT1A | F: CTCCGCCTGAGCCATGAAG | Sangon Biotech |
|  | R: CACCAGTGATGATGCCATTCT | Sangon Biotech |
| (Mouse) MCP1 | F: TTAAAAACCTGGATCGGAACCAA | Sangon Biotech |
|  | R: GCATTAGCTTCAGATTTACGGGT | Sangon Biotech |
| (Mouse) TNFα | F: CCCTCACACTCAGATCATCTTCT | Sangon Biotech |
|  | R: GCTACGACGTGGGCTACAG | Sangon Biotech |
| (Mouse) CXCL2 | F: CCAACCACCAGGCTACAGG | Sangon Biotech |
|  | R: GCGTCACACTCAAGCTCTG | Sangon Biotech |
| (Mouse) IL1β | F: GCAACTGTTCCTGAACTCAACT | Sangon Biotech |
|  | R: ATCTTTTGGGGTCCGTCAACT | Sangon Biotech |
| (Mouse) IL6 | F: TAGTCCTTCCTACCCCAATTTCC | Sangon Biotech |
|  | R: TTGGTCCTTAGCCACTCCTTC | Sangon Biotech |
| (Human) EMMPRIN | F: GAAGTCGTCAGAACACATCAACG | Sangon Biotech |
|  | R: TTCCGGCGCTTCTCGTAG | Sangon Biotech |
| (Human) CIDEA | F: TTATGGGATCACAGACTAAGCGA | Sangon Biotech |
|  | R: TGCTCCTGTCATGGTTGGAGA | Sangon Biotech |
| (Human) PPARγ | F: ACCAAAGTGCAATCAAAGTGGA | Sangon Biotech |
|  | R: ATGAGGGAGTTGGAAGGCTCT | Sangon Biotech |
| (Human) SREBP1C | F: CGGAACCATCTTGGCAACAGT | Sangon Biotech |
|  | R: CGCTTCTCAATGGCGTTGT | Sangon Biotech |
| (Human) FASN | F: AAGGACCTGTCTAGGTTTGATGC | Sangon Biotech |
|  | R: TGGCTTCATAGGTGACTTCCA | Sangon Biotech |
| (Human) PLD1 | F: GAGCCACGGGTAAATACCTCT | Sangon Biotech |
|  | R: CCGCGTGTCCAGATTTTCTATG | Sangon Biotech |
| (Human) DGAT1 | F: TATTGCGGCCAATGTCTTTGC | Sangon Biotech |
|  | R: CACTGGAGTGATAGACTCAACCA | Sangon Biotech |
| (Human) FATP2 | F: TACTCTTGCCTTGCGGACTAA | Sangon Biotech |
|  | R: CCGAAGCAGTTCACCGATATAC | Sangon Biotech |
| (Human) CD36 | F: GGCTGTGACCGGAACTGTG | Sangon Biotech |
|  | R: AGGTCTCCAACTGGCATTAGAA | Sangon Biotech |
| (Human) CXCL2 | F: CGCCCATGGTTAAGAAAATCA | Sangon Biotech |
|  | R: CCTTCTGGTCAGTTGGATTTGC | Sangon Biotech |
| (Human) IL1β | F: ATGATGGCTTATTACAGTGGCAA | Sangon Biotech |
|  | R: GTCGGAGATTCGTAGCTGGA | Sangon Biotech |
| (Human) IL6 | F: CCTGAACCTTCCAAAGATGGC | Sangon Biotech |
|  | R: TTCACCAGGCAAGTCTCCTCA | Sangon Biotech |

**Supplementary Table 2. List of the antibodies used**

| Antibody | Manufacturer | Cat No. | Clone no. | Citation |
| --- | --- | --- | --- | --- |
| Rabbit monoclonal antibody to EMMPRIN | Abcam | ab188190 | EPR18008-8 | AB_3674216 |
| Mouse monoclonal antibody to EMMPRIN | Proteintech | 66443-1-Ig | 1G12B5 | AB_2881813 |
| Rabbit polyclonal antibody to FASN | Proteintech | 10624-2-AP | polyclonal | AB_2100801 |
| Rabbit monoclonal antibody to ACC1 | Abcam | ab45174 | EP687Y | AB_867475 |
| Rabbit polyclonal antibody to CPT1A | Proteintech | 15184-1-AP | polyclonal | AB_2084676 |
| Rabbit polyclonal antibody to α-SMA | Bioss | bs-10196R | polyclonal | AB_2801647 |
| Rabbit polyclonal antibody to MCT1 | Proteintech | 20139-1-AP | polyclonal | AB_2878645 |
| Rabbit monoclonal antibody to UBA52 | zenbio | R27396 | R05-5D6 | AB_3714687 |
| Rabbit monoclonal antibody to UBA52 | GenulN | 5508 | 25GB1825 | AB_3714688 |
| Rabbit polyclonal antibody to UB | Cell Signaling Technology | 3933S | polyclonal | AB_2180538 |
| Rabbit monoclonal antibody to K63-UB | Cell Signaling Technology | 5621S | D7A11 | AB_10827985 |
| Rabbit monoclonal antibody to K48-UB | Cell Signaling Technology | 8081S | D9D5 | AB_10859893 |
| Rabbit monoclonal antibody to Pan-Lac | PTM Bio | PTM-1401RM | 9H1L6 | AB_2942013 |
| Mouse monoclonal antibody to FLAG-Tag | Proteintech | 66008-4-Ig | 8H6A10 | AB_2918475 |
| Mouse monoclonal antibody to FLAG-Tag | Share-bio | SB-AB0008 | Not found | AB_3674219 |
| Rabbit monoclonal antibody to COL1A1 | PTM Bio | PTM6219 | Not found | AB_3719449 |
| Rabbit monoclonal antibody to COL3A1 | Abcam | ab184993 | EPR17673 | AB_3719453 |
| Rabbit monoclonal antibody to TNFα | Abcam | ab183218 | EPR19147 | AB_3719452 |
| Rabbit monoclonal antibody to p-NFKB | Abcam | AB76302 | EP2294Y | AB_3719454 |
| Rabbit polyclonal antibody to NFKB | zenbio | 380172 | polyclonal | AB_3719448 |
| Rabbit polyclonal antibody to LC3 | Abmart | T55992F | polyclonal | AB_2929010 |
| Rabbit monoclonal antibody to P62 | Abmart | T55546F | Not found | AB_2936967 |
| Mouse monoclonal antibody to PCNA | PTM Bio | PTM-5187 | Not found | AB_3719451 |
| Mouse monoclonal antibody MCM2 | zenbio | 220635 | 2B5-H4-A9 | AB_3719450 |
| Rabbit monoclonal to ACTIN | Proteintech | 81115-1-RR | 4H1 | AB_2923704 |
